# The Emerging Role of microRNAs in Polyglutamine Diseases

**DOI:** 10.3389/fnmol.2019.00156

**Published:** 2019-06-19

**Authors:** Xiaoyu Dong, Shuyan Cong

**Affiliations:** Department of Neurology, Shengjing Hospital of China Medical University, Shenyang, China

**Keywords:** microRNAs, polyglutamine diseases, biomarker, neurodegeneration, therapy

## Abstract

MicroRNAs (miRNAs) are small non-coding molecules that regulate a large amount of post-transcriptional repressor genes by recognizing semi-complementary target sequences that are normally located in the 3′ UTR of the mRNA. Altered expression of miRNA has been related to several pathological processes, including polyglutamine (Poly Q) diseases. Specific expression patterns in the circulating fluids and brain parenchyma have been speculated as potential biomarkers for Poly Q disease diagnosis and prognosis. Several miRNAs have been consistently identified in diseases including Huntington’s disease (HD) and spinocerebellar ataxia (SCA). In our review, we describe the emerging role of miRNAs in Poly Q diseases and provide an overview on general miRNA biology, implications in pathophysiology, and their potential roles as future biomarkers and applications for therapy.

## Introduction

Polyglutamine (Poly Q) diseases are characterized by trinucleotide repeat amplification within a gene, which results in the formation of Poly Q peptides, selective neuronal degeneration and death in neurodegenerative diseases (Liu et al., 2018). To date, there are at least nine known Poly Q diseases, including Huntington’s disease (HD), spinobulbar muscular atrophy (SBMA), dentatorubral-pallidoluysian atrophy (DRPLA), and six spinocerebellar ataxias (SCAs; He et al., 2010). Although different Poly Q diseases involve different pathogenic genes, a large amount of studies have shown a common pathogenesis mechanism related to “gain of toxic functions.” That is, the pathogenic Poly Q proteins cause selective neuronal toxicity; accumulation of Poly Q-mutant proteins and formation of neuronal intranuclear inclusions (NIIs) are core elements of the pathogenic process (Yushchenko et al., 2018). Hypotheses regarding possible pathogenic mechanisms include: (1) protein misfolding; (2) gene transcription and expression imbalance; (3) intracellular protein homeostasis failure; and (4) mitochondrial dysfunction.

In recent years, microRNAs (miRNAs) have been confirmed to play important roles in both physiological and pathological conditions of many brain-related functions (Saraiva et al., 2017). Abnormal miRNA expression patterns have been proposed to be related to the etiology and progression of Poly Q diseases. Several studies have also shown that increases or decreases in expression levels can be used as potential diagnostic biomarkers or to ameliorate neurodegenerative processes and promote endogeneous regeneration.

In this review article, we introduce the canonical miRNA biogenesis pathway and miRNA function and describe the most relevant brain-specific miRNAs associated with neurodegenerative processes in Poly Q disease. Potential biomarkers involved in Poly Q disease diagnosis and prognosis and the advantages and limitations of using miRNA-based therapeutic strategies will also be highlighted.

## MiRNA Basics

MiRNAs are a novel class of small (~21 nucleotides), single-stranded, non-coding RNAs that act as post-transcriptional regulators of gene expression (Goodall et al., 2013). MiRNAs function by binding to the 3′-UTR of mRNA targets and recruiting the RNA-induced silencing complex (RISC) to inhibit target expression (Lau and de Strooper, 2010). The mechanism of how RISC inhibits the expression of bound mRNA targets remains unclear (Filipowicz et al., 2008). Inhibition in translation at the initiation step (Mathonnet et al., 2007) or deadenylation of targets followed by mRNA decay may take part in the process (Wakiyama and Yokoyama, 2014). To date, more than 10,000 miRNA sequences have been included in miRBase^1^, of which 721 have been identified in the human genome, and approximately 30% are encoded within human protein-encoding genes (Carroll et al., 2011). Primary miRNA (pri-miRNA) genes are normally transcribed by RNA polymerase II and can generate an incomplete stem-loop structure of several thousand bases in length (Yin et al., 2015). Pri-miRNA is further processed by the microprocessor complex in the nucleus and can generate precursor miRNAs (pre-miRNAs), which are characterized by a stem-loop structure of ~70 nucleotides. After export from the nucleus by Exportin 5/Ran, pre-miRNAs are generally cleaved by Dicer to generate mature miRNAs (Winter et al., 2009; Lau and de Strooper, 2010).

MiRNAs play an important role in various physiological processes in the nervous system. They are involved in regulating neurodevelopment, proliferation, plasticity, and memory and are closely associated with the development of a variety of nervous system disorders (Snyman et al., 2017). MiRNA expression is usually regulated by epigenetic mechanisms, including histone modification, DNA methylation, or by enzymes that stabilize mature miRNAs (Femminella et al., 2015). Studies have shown that there may be more than 1,000 types of miRNAs expressed in the human brain and miRNA expression in the nervous system has regional, tissue and spatiotemporal specificity. In particular, miR-10b-5p, miR-145-5p, miR-378c, and miR-217 are mainly expressed in the prefrontal cortex, miR-205 in the frontal cortex and miR-21-3p, miR-224, and miR-373-3p are mainly expressed in the substantia nigra. MiR-134 is involved in the formation of dendritic spines, while miR-124 and miR-132 participate in axon growth. As some studies have pointed out, these small RNAs have been identified as stable in blood and cerebrospinal fluid (CSF; Wu et al., 2016). Techniques such as quantitative PCR and microarrays are being applied to measure miRNA levels in biofluids. Microarray analysis is generally used as a non-targeted approach to analyze multiple miRNAs simultaneously, while PCR is more accurate and sensitive for validating results obtained from microarray analysis (Huang, 2017).

Recently, aberrant expression or deregulation of miRNAs has been found to play a major role in the pathogenesis of several Poly Q diseases (Shah et al., 2018). Their potential as diagnostic and prognostic biomarkers is gradually being recognized and applied in clinical practice. Gene-silencing methods that target miRNAs have been used to treat several Poly Q diseases in animal models.

## MiRNA in Poly Q Disease

### MiRNAs in HD

In Europe and North America, the incidence of HD per 100,000 people is 5%–10% (Graham et al., 2018). CAG trinucleotide repeats within the huntingtin gene result in abnormal accumulation of the misfolded huntingtin (HTT) protein in intranuclear inclusions and lead to progressive loss of striatal neurons (Lau and de Strooper, 2010), which are the main causative factors of the disease (Hu et al., 2010). The clinical characteristics of HD are dystonia, chorea, and cognitive or psychiatric disorders. There is currently no effective treatment and patients usually die 10–20 years after illness onset (Jacobsen et al., 2010). By applying RNA sequencing, microarray, and qRT-PCR techniques, aberrant miRNA expression in HD has been reported in a large number of studies using different human samples. Chang et al. (2017) found the expression of a set of miRNAs was dysregulated in Brodmann’s area four in HD patients including miR-218, miR-196a, and miR-486, which are highly expressed in the pathological region, and miR-132 miR-9, miR-124a, miR-29b and miR-22, which show low expression in HD patients. Martí et al. (2010) also found that miR-100, miR-16, miR-151-3p, miR-219-2-3p, miR-451, miR-27b, and miR-92a were up-regulated in the striatum and frontal cortex of HD patients, while miR-128, miR-222, miR-139-3p, miR-382, miR-483-3p and miR-433 were down-regulated in the diseased tissues. In recent decades, a great number of HD cell model and animal studies have shown that miRNAs may affect the pathogenesis, progression, and prognosis of patients through various pathways (Johnson and Buckley, 2009). In a monkey model, Kocerha et al. (2014) pointed out that miR-128 was down-regulated in the brain of pre- and post-symptomatic HD monkeys; by suppressing HIP-1, HTT and SP-1, they concluded that miR-128 may play a pivotal role in HD pathogenesis. Repressor element-1 silencing transcription factor (REST) is a zinc finger protein that binds to the corresponding neuron-restricted silencing elements (NRSEs) and deacetylates histones to inhibit the expression of some neurogenic genes (Soldati et al., 2013). Poly Q expansion of mutant HTT protein can inhibit the interaction between REST and the HTT protein, thereby promoting REST aggregation in the nucleus of HD patients and inhibiting the expression of related genes (Wu and Xie, 2006). Several studies have found that REST is regulated by a variety of miRNAs including miR-29a, miR-29b, miR-132, and miR-135b (Reed et al., 2018). Johnson et al. (2008) demonstrated that miR-9, -29a, -29b, -124a, -132, -135b, -139, -203, -204, -212, -330, and -346 were also associated with REST expression. MiRNAs may also have neuroprotective functions. Jovicic et al. (2013) identified miR-22 as a potentially neuroprotective miRNA in HD by regulating several targets such as REST corepressor 1 (Rcor1), histone deacetylase 4 (HDAC4) and regulator of G-protein signaling 2 (Rgs2). MiR-196a may ameliorate cytotoxicity and the cellular phenotype in transgenic HD monkey neuronal cell models.

In the following section, we will review some of the miRNAs that may be associated with HD pathology and have been consistently identified as dysregulated in HD (Table 1; Chen J. et al., 2018).

**Table 1:** MicroRNAs (miRNAs) most commonly associated with Huntington’s disease (HD).

MiRNA	Role in HD pathophysiology	Evidence in HD patients	References
MiR-22	Regulates multiple mRNAs involved in the pathogenesis of HD; targets include HDAC4, REST, Rgs2	Down-regulated in the brain	Xiong et al. (2010) and Jovicic et al. (2013)
MiR-132	Negative correlation with p250GAP; targets include p250GAP, MeCP2, REST	Down-regulated in the cortices	Klein et al. (2007) and Chen D. et al. (2018)
MiR-124	Crucial regulator for neuronal differentiation in neurodegeneration; targets include SOX9, PTB1, PGC1	Down-regulated in the brain	Makeyev et al. (2007) and Liu et al. (2015)
MiR-196a	Suppresses mutant HTT expression at the mRNA and protein levels; targets include mutant HTT, ANX1A, BDNF	Up-regulated in the prefrontal cortex	Moumné et al. (2013), Tan L. et al. (2015) and Kunkanjanawan et al. (2016)
MiR-10b-5p	Targets HTT by binding to 3′ UTR sites and reducing expression; targets include mutant HTT, BDNF, CREB1	Up-regulated in the prefrontal cortex	Hoss et al. (2015a,b), Jamwal and Kumar (2015) and Lewis et al. (2005)
MiR-146a	Regulator of inflammation-related mRNA, acts as an inflammatory response repressor in the CNS; targets include mutant HTT, TBP	Up-regulated in the brain	Sonkoly et al. (2008), Sinha et al. (2010) and Laprairie et al. (2019)

MiR-22 was originally identified in HeLa cells (an immortal cell line derived from cervical cancer cells), but was later shown to be ubiquitously expressed in various tissues (Xiong et al., 2010) and down-regulated in HD brain tissue (Jovicic et al., 2013). Using TargetScan, miR-22 was found to target multiple mRNAs implicated in HD pathogenesis; among the targets is HDAC4, which has been shown to reverse histone acetylation and plays a neuroprotective effect in animal and cellular models of HD (Harding and Tong, 2018). The REST pathway is hyperactive in HD, which leads to large-scale repression of neuronal genes in affected neurons (Zuccato et al., 2007). Seredenina et al. (2011) reported that decreased expression of Rgs2 in striatal neurons has a protective effect in cell models of HD by reducing activation of extracellular-signal-regulated kinase (ERK). Although the specific mechanism is not yet elucidated, miR-22 could regulate HDAC4, REST corepressor 1 (Rcor1) and Rgs2 to achieve neuroprotection and inhibit neurodegeneration (Jovicic et al., 2013).

MiR-132 is a key component of the activity-dependent gene regulatory response in neuronal cells (Chen D. et al., 2018). Vo et al. (2006) determined that miR-132 promotes neuronal outgrowth and sprouting by decreasing p250GAP levels. Another target for miR-132 is methyl CpG binding protein 2 (MeCP2). Failure to regulate MeCP2 levels is connected to neurological disorders (Klein et al., 2007). As one of the most important growth factors that preserve neural cell growth and survival, BDNF has been identified dysregulation in HD patients. MeCP2 increases BDNF levels in the brain, which in turn increases transcription from the miR-132 cluster. Thus, miR-132 dysregulation might contribute to HD progression by impairing neurogenesis and affecting BDNF balance in the diseased brain (Conaco et al., 2006). A rise in miRNA-132 levels will decrease MeCP2 levels and restore the balance. Failure to regulate MeCP2 levels is connected to neurological disorders (Klein et al., 2007). REST has been reported to repress BDNF, which is a CREB-dependent activator of miR-132 and directly repress miR-132 transcription. Thus, REST could effectively repress the expression of miR-132 in two independent pathways by an effective repressive feed-forward loop (Johnson and Buckley, 2009; Bicker et al., 2014).

MiR-124 is a neuronal-specific miRNA and is thought to be a crucial regulator of neuronal differentiation in neurodegeneration (Liu et al., 2015). SRY-related HMG box transcription factor 9 (SOX9) is a physiological target of miR-124; repression of SOX9 mediated by miR-124 is essential for neuron formation from neural stem cells (Cheng et al., 2009). MiR-124 could promote the differentiation process by facilitating neuron-specific splicing of mRNAs through repression of polypyrimidine tract binding protein (PTB1) in developing neurons. Thus, a decrease in the level of miR-124 may promote the accumulation of non-neuronal splice isoforms in neurons (Makeyev et al., 2007; Johnson and Buckley, 2009). As a presumed target of miR-124, Cyclin A2 (CCNA2) is increased in mutant STHdh (Q121)/Hdh (Q121) cells. Predictably, decreased expression of miR-124 would increase CCNA2 levels in animal and cell models of HD and would be resulted in cell cycle deregulated in the HD cell model (Das et al., 2013). In contrast, miR-124 overexpression would up-regulate BDNF and peroxisome proliferator-activated receptor gamma coactivator 1 (PGC1) expression and down-regulate SOX9 expression in the striatum of R6/2 mouse brain, which implies that miR-124 slows down the HD progression (Meza-Sosa et al., 2012).

Several recent researches have confirmed the protective function of miR-196a in HD disease (Moumné et al., 2013). Tan L. et al. (2015) suggested that miR-196a suppresses mutant HTT expression at the protein and mRNA levels. Moreover, the inhibition was not resulted by binding of miR-196a to the 3′-UTR of mutated HTT directly but occurred through inhibiting the synthesis of protein and partly through enhancing the degradation of protein (Tan L. et al., 2015). MiR-196a also targets ANX1A to promote cell survival and reduce apoptosis. Over-expression of miR-196a could enhance *BDNF* expression, which benefits neural cell survival in HD animal models (Kunkanjanawan et al., 2016).

In recent years, MiR-10b-5p has been reported to be significantly up-regulated in the HD brain (prefrontal cortex) and data suggest that this change may occur pre-symptomatically. As one of the targets of miR-10b-5p (Jamwal and Kumar, 2015), BDNF down-regulation in HD brains might be mediated through up-regulation of miR-10b-5p, which has a binding target within the 3′-UTR of the BDNF transcript (Varendi et al., 2014). CREB1 is another target of this miRNA. The 3′-UTR of CREB1 also harbors a miR-10b-5p binding site and reduced CREB1 expression has been reported in HD (Lewis et al., 2005). Moreover, miR-10b-5p presumably targets HTT by binding to two 3′-UTR sites (both 7mer-1A seeds, positions 2,742–2,748 and 3,301–3,307) and could decrease HTT expression (Ritchie, 2017). Given the stability of miR-10b-5p in serum, it could be considered an accessible biomarker for stage of disease, rate of progression and other important clinical HD characteristics (Hoss et al., 2014).

MiR-146a is primarily involved in regulating inflammation and other processes that have important functions in the innate immune system (Sonkoly et al., 2008). Recruitment of the basal transcription factor TBP (tata binding protein) to disruption of TBP structures by HTT or mutant HTT aggregates, may compromise its function (Laprairie et al., 2019). MiR-146a could directly regulate TBP expression; decreased expression of miR-146a would lead to high TBP expression levels and further affect cellular responses in HD cell models and contribute to the pathogenesis of HD (Sinha et al., 2010).

### MiRNA in SCAs

SCAs are chronic progressive neurological disorders in which cerebellar ataxia is the most prominent symptom (Roshan et al., 2014; van der Stijl et al., 2017). Of more than 40 different SCA types (Durr, 2010), there are at least six SCAs (SCA-1, 2, 3, 6, 7, and 17) that are associated with Poly Q diseases (Sinha et al., 2018). The expression of CAG repeats of abnormal length in the open reading frame (ORF) of the ataxin (*ATXN*) genes are the common denominator. Specific linkages between miRNA regulation and CAG repeat-dependent SCAs have been described in some studies (Chau and Kalsotra, 2015; Table 2).

**Table 2:** Summary of current links between miRNA regulation and spinocerebellar ataxia (SCAs).

Disease	miRNA change	Role in SCA pathophysiology	Evidence in experimental models or in SCA patients	References
Spinocerebellar ataxia type 1 (SCA1)	MiR-19a, miR-101, miR-130a, miR-144, miR-150 up-regulated	MiR-19a, miR-101, miR-130a and miR-144 inhibit ATXN1 translation; miR-150 targets Rgs8, Vegfa	SCA1 the brain of patients; in a mouse model	Lee et al. (2008), Rodriguez-Lebron et al. (2013), Koscianska and Krzyzosiak (2014), Keiser et al. (2016) and Cvetanovic et al. (2017)
Spinocerebellar ataxia type 2 (SCA2)	MiR-12 up-regulated	Ataxin-2 protein interacts with Ago1to impair the repressive activity of miR-12	In a *Drosophila* model	McCann et al. (2011)
Spinocerebellar ataxia type 3 (SCA3)	MiR-34b up-regulated, miR-29a, miR-25, miR-125b, miR-9, miR-181a, and miR-494 down-regulated	MiR-25, miR-9, miR-181a, and miR-494 down-regulate mutant ATXN3; miR-9, miR-181a, and miR-494 target DGCR8, Dicer, and FMR1	SCA3 in the brain of patients	Bettencourt and Lima (2011), Huang et al. (2014), Shi et al. (2014) and Carmona et al. (2017)
Spinocerebellar ataxia type 6 (SCA6)	MiR-3191-5p up-regulated	Targets CACNA1A IRES and attenuates α1ACT translation to prevent ataxia and motor deficits	In a mouse model	Miyazaki et al. (2016)
Spinocerebellar ataxia type 7 (SCA7)	MiR-124 down-regulated	Represses ataxin-7 translation	In a mouse model	Tan J. Y. et al. (2015)
Spinocerebellar ataxia type 17 (SCA17)	MiR-29a/b down-regulated	Fulfills the anti-apoptotic role by regulating BACE1	In a mouse model	Roshan et al. (2012) and Tan J. Y. et al. (2015)

SCA1 is a dominantly inherited neurodegenerative disease caused by repeated expansions of Poly Q in ataxin-1 (Rodriguez-Lebron et al., 2013). Lee et al. (2008) revealed that miR-19a, miR-101, and miR-130a co-regulate the 3′-UTR of *ATXN1* by inhibiting *ATXN1* translation. Using a reporter assay, miR-19a, -101, and -130a were shown to down-regulate *ATXN1* expression (Lee et al., 2008). MiRNA-mediated post-transcriptional regulation of the *ATXN1* gene may modulate SCA1-associated neuropathology by affecting levels of ATXN1 protein (Koscianska and Krzyzosiak, 2014).

MiR-144 was predicted to be related to SCA1 in a genome-wide microarray analysis (Persengiev et al., 2011). MiR-144 considerably decreased endogenous ATXN1 protein levels. In addition, activation of miR-144 reduced the cytotoxic effects of the expanded mutATXN1 and miRNA deregulation may be involved in SCA1 development. It was also demonstrated that increased miR-150 levels influenced SCA1 pathogenesis by suppressing Rgs8 expression and contributed to the reduction of Vascular Endothelial Growth Factor A (VEGFA) in cerebellar Purkinje neurons in a mouse model of SCA1 (Rodriguez-Lebron et al., 2013; Cvetanovic et al., 2017).

SCA2 is an autosomal dominant hereditary neurodegenerative disorder. It is caused by a repeated amplification of CAG in the coding region of the *ATXN2* gene, resulting in an abnormally long poly Q tract expression in the ataxin-2 protein and acquired neurotoxicity leading to neuronal loss in the brainstem, cerebellum, brain cortex and spinal cord. At present, little research has been done on the role of miRNAs in the pathogenesis of SCA2. McCann et al. (2011) showed that ataxin-2 might be required for miRNA functioning in *Drosophila*; it was also shown the ataxin-2 protein impairs the repressive activity of miR-12 by interacting with Argonaute 1 (Ago1).

SCA3/Machado-Joseph disease (MJD) is caused by CAG repeated amplification of the *ATXN3* gene (Shi et al., 2014). Using miRCURY™ LNA Array followed by qRT-PCR validation, Shi et al. (2014) found that miR-34b expression was significantly elevated, whereas miR-25, miR-29a and, miR-125b were down-regulated in SCA3 patient serum. Regarding molecular mechanisms, miR-25 and miR-125b may bind the 3′-UTR of *ATXN3* and regulate the *ATXN3* expression. Moreover, miR-25 and miR-125b expression were also associated with disease progression (Shi et al., 2014). MiR-25 reduces mutant ATXN3 protein levels by interacting with the 3′-UTR of the *ATXN3* mRNA, thereby decreasing early apoptosis, increasing cell viability and alleviating the accumulation of mutant ATXN3 protein aggregates in SCA3/MJD cells (Huang et al., 2014). Carmona et al. (2017) revealed that miR-9, miR-181a, and miR-494 were significantly down-regulated in SCA3/MJD based on *in vivo* and *in vitro* models and gene expression analysis. Down-regulation of DGCR8, Dicer, and FMR1 may be responsible for miRNA dysregulation. Dual luciferase assay confirmed that miR-9, miR-181a and miR-494 bind directly to the *ATXN3* 3′-UTR and down-regulate its expression. Furthermore, miR-181a and miR-494 down-regulate muttATXN3 expression either by inhibiting translation or by inducing the degradation of its mRNA, while miR-9 acts mainly through translational inhibition *in vitro* (Carmona et al., 2017).

SCA6 is a dominant hereditary neurodegenerative disorder characterized by slowly progressive ataxia and Purkinje cell degeneration. It is caused by repeated amplification of Poly Q in the second CACNA1A gene product, α1ACT; expression of α1ACT is controlled by the internal ribosomal entry site (IRES) present within the coding region of CACNA1A (Miyazaki et al., 2016). MiR-3191-5p targets the CACNA1A IRES and preferentially attenuates IRES-driven α1ACT translation, in an Ago4-dependent manner, to cause Purkinje cell degeneration. Viral delivery of miR-3191-5p can rescue the disease phenotype by modulating the cellular IRES activity in a mouse model (Miyazaki et al., 2016).

SCA7 is a rare inherited neurodegenerative disorder caused by an in-frame repeated CAG expansion in the first exon of the ataxin-7 gene (*ATXN7*, David et al., 1997). Translation of the mutated *ATXN7* allele results in expansion of poly Q tracts, the formation of protein aggregates and decreases the protein activity (Martin et al., 1999). MiR-124 was determined to repress ataxin-7 mRNA translation by modulation of a conserved long-ncRNA-mediated negative feedback loop. By inhibiting STAGA activity, the loop is disrupted by the poly Q-expanded ataxin-7, resulting in decreased miR-124 transcription, and followed by increased poly Q-expanded ataxin-7 translation in the retina and cerebellum (Das et al., 2013; Tan J. Y. et al., 2015; van der Stijl et al., 2017).

SCA17 is caused by the expansion of a repeated Poly Q in the *TBP* gene and is characterized by intranuclear protein aggregation and selective loss of cerebellar neurons (Roshan et al., 2017). MiR-29a/b has been found down-regulation in a SCA17 cellular model by qRT PCR. MiR-29a/b down-regulation may lead to an increased expression of beta-site amyloid precursor protein cleaving enzyme 1 (BACE1), Bcl 2 homologous antagonist (BAK), p53 upregulated modulator of apoptosis (PUMA), increased cytochrome c release and apoptosis restoration of miR-29a/b in the pathogenic poly Q background reduced expression of BACE1 (Das et al., 2013). The miR-29a/b-BACE1 regulatory interaction may be part of the molecular mechanism that leads to neuronal cell death in SCA17 (Roshan et al., 2012).

### MiRNAs in SBMA

SBMA is an adult-onset neuromuscular disease caused by repeated expansion of poly Q in the androgen receptor (AR; La Spada et al., 1991). MiR-298 downregulates the levels of AR mRNA and protein when transfected into cells overexpressing wild and mutant type AR and fibroblasts derived from patients with SBMA. Furthermore, miR-298 could counteract AR toxicity by binding directly to the 3′-UTR of the human AR transcript *in vitro* (Pourshafie et al., 2016).

### MiRNAs in DRPLA

A poly Q expanded mutant Atrophin-1 protein is the pathogenic protein of DRPLA. Atrophin-1 mRNA is one of the direct targets of miR-8. MiR-8 mutant phenotypes can be attributed to an increase in atrophin activity, which leads to an increase in brain apoptosis and behavioral defects (Huang et al., 2010). Deletion of miR-8 was found to increase expression of Atrophin-1 in a Drosophila model, thereby causing degeneration of nerve cells and impaired motor function. In addition, miR-200b and miR-429 are identical to miR-8 in the seed region, which permits miR-200b and miR-429 to recruit HDACs and serve as a transcriptional corepressor. Therefore, they could target the 3′-UTR of human Atrophin-1 to contribute to the pathogenesis of DRPLA (Karres et al., 2007).

## MiRNAs as Biomarkers in Poly Q

The diagnosis of probable Poly Q diseases is based on clinical and neuropsychological assessments and can be confirmed by genetic testing. However, early treatment is crucial in these diseases and there is an urgent need for easily accessible biomarkers to aid in early diagnosis. Moreover, with several therapeutic approaches in development for Poly Q disease, biomarkers could also be utilized to monitor disease progression and responses to therapy (Table 3).

**Table 3:** Aberrant expression of miRNAs as biomarkers in Poly Q diseases.

miRNAs	Source	Changes in expression	Sample size	References
In HD				
MiR-30d-5p, miR-877-5p, miR-425-5p, miR-223- 3p, miR-128,	plasma	up-regulation	15P, 7C	Díez-Planelles et al. (2016)
miR-22-5p, miR-338-3p, miR-130b-3p, miR-628-3p, miR-223-5p, miR-361-5p, miR-942 and miR-222-3p
MiR-10b-5p, miR-486-5p, miR-132-3p, and miR-363-3p	brain	up-regulation	28P, 36C	Hoss et al. (2015a)
MiR-10b-5p and miR-486-5p	plasma	up-regulation	26P, 8C	Hoss et al. (2015b)
MiR-34b	plasma	up-regulation	27P, 12C	Gaughwin et al. (2011)
MiR-9	plasma	down-regulation	44P, 28C	Chang et al. (2017)
miR-100, miR-151-3p, miR-16, miR-219-2-3p, miR-27b, miR-451, and miR-92a	brain	up-regulation	5P, 5C	Martí et al. (2010)
MiR-128, miR-139-3p, miR-222, miR-382, miR-433,	brain	down-regulation	5P, 5C	Martí et al. (2010)
and miR-483-3p
MiR-520f-3p, miR-135b-3p, miR-4317, miR-3928-5p,	CSF	up-regulation	45P, 15C	Reed et al. (2018)
miR-8082, miR-140-5p
In SCA3				
MiR-34b	plasma	up-regulation	35P, 25C	Shi et al. (2014)
MiR-29a, miR-25 and miR-125b	plasma	down-regulation	35P, 25C	Shi et al. (2014)
In SCA7				
Hsa-let-7a-5p, hsa-let7e-5p, hsa-miR-18a-5p, and	plasma	up-regulation	35P, 17C	Borgonio-Cuadra et al. (2019)
hsa-miR-30b-5p

*CSF, Cerebrospinal fluid; P, patients; C, Controls*.

It has been reported that miRNAs are present in various biofluids, such as saliva, blood, urine, tears, breast milk, bronchial secretions, amniotic fluid and CSF (Weber et al., 2010). The stability and abundance of circulatory miRNAs in biofluids are the main factors in clinical application as potential diagnostic and progression biomarkers (Li et al., 2018). There may be five possible miRNA transportation mechanisms into the extracellular circulation. MiRNAs may: (1) form a complex with the Ago2 protein; (2) be packaged within exosomes; (3) be encapsulated within micro-vesicles; (4) be bound to high-density lipoprotein (HDL) particles in non-vesicle form; or (5) accumulate in apoptotic bodies (Kumar et al., 2017). However, the details of these mechanisms need to be further clarified (Chen et al., 2012).

In this section, we review the findings of the most significant studies to investigate the potential role of miRNAs as biomarkers in Poly Q diseases.

Mature miRNA levels were determined from 752 plasma samples obtained from 15 symptomatic patients with 40–45 CAG repeats in the *HTT* gene and seven healthy matched controls (Kumar et al., 2017). Alterations in a total of 168 plasma miRNAs were identified in symptomatic patients with a significant up-regulation of 13 miRNAs (miR-30d-5p, miR-877-5p, miR-425-5p, miR-223-3p, miR-128, miR-22-5p, miR-338-3p, miR-130b-3p, miR-628-3p, miR-223-5p, miR-361-5p, miR-942 and miR-222-3p; Díez-Planelles et al., 2016). In addition, four miRNAs (miR-132-3p, miR-363-3p, miR-10b-5p and miR-486-5p) showed significant genome-wide changes in HD brain tissue and were abundant in both brain and blood. Plasma samples from 26 HD patients (compared to eight controls) had increased miR-10b-5p and miR-486-5p levels, whereas the levels of miR132-3p, though not significant, were lower (Hoss et al., 2015a). MiR-10b-5p was subsequently confirmed to be related to the age of onset of the disease (Hoss et al., 2015b; Ghatak and Raha, 2018). Similar findings were reported, in which 11 pre-manifest HD, eight early HD, eight moderate HD, and 12 control subjects were included (Gaughwin et al., 2011). The results revealed that miR-34b and miR-1285 were up-regulated in the plasma of HD patients and, most interestingly, the levels of miR-34b in the plasma of pre-manifest HD patients were significantly increased (Ridolfi and Abdel-Haq, 2017).

In recent years, an increasing number of miRNA profiles have been used in SCA research. Shi et al. (2014) searched for different plasma levels of miRNAs from 35 SCA3 patients and 25 controls. Plasma miR-34b levels were significantly elevated, whereas miR-25, miR-29a, and miR-125b levels was down-regulated. Additional research showed that miR-25 and miR-125b expression levels were related to disease progression (Langfelder et al., 2018). In SCA7 patients, a signature of four miRNAs (hsa-let-7a-5p, hsa-let7e-5p, hsa-miR-18a-5p, and hsa-miR-30b-5p) were correlated with disease severity and stage. These miRNAs could help to distinguish early-onset from adult-onset disease and to identify novel cellular processes associated with SCA7 (Borgonio-Cuadra et al., 2019).

There is increasing evidence that changes in miRNA levels in serum/plasma, CSF and other biological fluids are associated with certain biological conditions, such as HD and SCA3. Novel informative biomarkers could provide more information about the development and progression of these diseases and facilitate observation of disease progression and the effects of treatment. However, a more refined understanding of the mechanisms regarding how circulating miRNAs change as disease development and progression will be required to effectively use miRNAs in biomarker applications (Kumar et al., 2017). Additionally, clinical studies that include more patients at different stages of the disease will be essential.

MiRNAs play important roles in regulating gene expression in different cellular processes and disease pathogenesis. By interacting with complementary mRNAs at the post-transcriptional level to affect most mechanisms, deregulation of miRNAs is becoming a contributor to neurodegeneration (Miniarikova et al., 2016). Studying miRNAs associated with neurodegenerative diseases might also provide strategies for innovative therapies. Since the first phase 1 miRNA replacement therapy clinical trial was posted on Clinical Trials.gov (Wen, 2016), miRNAs have been extensively studied as promising therapeutic tools in a variety of diseases, including neurodegenerative diseases.

Poly Q diseases are monogenic dominantly inherited disorders with complex etiologies. To date, a large variety of potential therapeutic strategies have been applied in HD and other Poly Q diseases. Attempts include delivery of neurotrophic factors, mitochondrial function normalization, activation of neuronal stem cells and mutant gene targeting (Miniarikova et al., 2016). The most promising treatment methods at present are directed at lowering mutant mRNA levels.

Interfering with the expression of pathogenic mRNA by synthesizing antisense oligonucleotides (ASOs), small interfering RNAs (siRNAs), single-stranded RNAs (ssRNAs), the expressions of short hairpin RNAs (shRNAs) or artificial miRNAs have been widely explored. Although ASO and siRNA strategies have made progress in the treatment of some Poly Q diseases (Stiles et al., 2012; Southwell et al., 2014), efficacy and safety still need to be confirmed when sufficient ASOs and siRNAs are applied to the human brain. Moreover, the half-lives of ASOs and siRNAs are relatively short, requiring repeated administration, which impose a clinical burden on patients.

Previous studies have indicated that siRNA, shRNA, or miRNA expression can significantly silence the *HTT* gene by delivering with adeno-associated viral (AAV) vectors in murine HD models. Consistently, a miRNA-based expression system was suggested as a promising approach for HD treatment due to lower toxicity (Borel et al., 2011). Miniarikova et al. (2016) evaluated three different approaches for *HTT* gene silencing (including mtHTT and wtHTT) using expressed artificial miHTTs (miRNAs targeting *HTT*) in human and mouse HD models. Their findings considered miHTTs to be the best treatment strategy for further development. Sufficient inhibition of mtHTT, while maintaining wtHTT at appropriate levels, was achieved in several researches (Boudreau et al., 2009; McBride et al., 2011). Furthermore, bilateral striatal injections of AAV9-GFP-miR^Htt^ vectors lowered mRNA expression of HTT by >50% in an HD mouse model. This treatment was well tolerated; it did not appear to eliminate HTT expression completely and reduced HTT levels may be sufficient to significantly slow disease progression (Holmans et al., 2017).

Similar treatment strategies have also been studied in SCA1. Keiser et al. (2016) tested an artificial miRNA targeting the mouse *ATXN1* gene (in a knock-in model) and human *ATXN1* (in a transgenic model) utilizing AAV serotypes 2/1 and 2/5, respectively. Delivery of AAVs encoding RNA interference (RNAi) sequences to the cerebella of SCA1 mice resulted in widespread cerebellar Purkinje cell transduction and improved behavioral and histological phenotypes. This approach induced inhibition of ataxin-1 in the cerebellar cortex and brainstem neurons and may be sufficient for SCA1 treatment.

SCA3/MJD is the most common of the SCAs. Recently, two studies used artificial miRNA mimics to target the 3′-UTR of human *ATXN3*. A recombinant AAV was used to deliver the mimics to the cerebellum of transgenic mice expressing the full human disease gene (SCA3/MJD84.2 mice) and it was shown that anti- ATXN3 miRNAs effectively phenocopy the suppression of human *ATXN3* expression in SCA3/MJD84.2 mice (Rodríguez-Lebrón et al., 2014). However, despite significant knockdown of cerebellar ATXN3 and apparent tolerance of the constructs, the mice still developed motor phenotypes (Costa et al., 2013).

In addition to cerebellar neurodegeneration and ataxic symptoms, SCA7 patients usually lose visions (Garden and La Spada, 2008). Using non-allele-specific artificial miRNAs targeting ataxin-7 (*ATXN7*) injected into either the dorsal cochlear nucleus (DCN) or the eye of SCA7 mice, Ramachandran et al. (2014a) delivered AAV2/1 to the DCN and found relief of the motor phenotypes, and improvement of cerebellar Purkinje cell dendrites. Subsequently, it was verified that injection of AAV2/1 to the retina of SCA7 mice could achieve more than 50% inhibition of ATXN7 in the retina without side effects in retinal functioning and further confirmed the safety of long-term knockout of ATXN7 in the cerebellum and brainstem (Ramachandran et al., 2014a,b).

Some progress has also been made in the treatment of SBMA. Expression of miR-196a enhanced the decay of the AR mRNA by silencing CUGBP (an Elav-like family member 2 encoded by the *CELF2* gene), which directly acted on AR mRNA and enhanced its stability. Furthermore, the early intervention of the AAV vector on miR-196a improved the SBMA phenotypes in a mouse model (Miyazaki et al., 2012). Moreover, overexpression of miR-298 in SBMA mice by using a recombinant AAV9, delivered by just a single tail-vein injection, could induce sustained and extensive overexpression of skeletal muscle and motor neurons and lead to improvement of the neuromuscular phenotype in mice (Pourshafie et al., 2018).

We conclude that suppression of expanded mutant proteins in Poly Q diseases can have a significant effect on disease progression in animal models (Keiser et al., 2016). However, more researches should be conducted on the pharmacokinetics of miRNA to understand the threshold copies of miRNA that should be replaced or inhibited in each disease state. Specific miRNA carriers also should be designed for long term gene expression, as knockout in the CNS remains a tremendous challenge, that is as yet to be overcome (Wen, 2016).

## Conclusions

MiRNAs have emerged as a novel class of RNA molecules that play a crucial role in the pathological events of neurodegenerative diseases. It is important to note that many miRNAs have been identified to be significantly deregulated in Poly Q diseases, some of which have been consistently identified as Poly Q disease-specific. Circulating miRNAs are promising biomarkers for Poly Q diseases and for monitoring disease progression and therapy response. Although miRNAs are considered stable in biological fluids, variability still exists for numerous reasons. A more refined understanding of the mechanisms regarding how circulating miRNAs change as the disease development and progression will be required for accurate miRNA biomarker applications. Since *in vitro* and *in vivo* researches have indicated a dramatic effect on suppression of mutant proteins, developing efficient miRNA delivery techniques holds great promise for potential therapeutic approaches in the treatment of poly Q diseases.

## Author Contributions

This manuscript was primarily written by XD. Tables were produced by XD and SC. SC contributed to editing the review and contributed to the review revision. All authors read and approved the final manuscript.

## Conflict of Interest Statement

The authors declare that the research was conducted in the absence of any commercial or financial relationships that could be construed as a potential conflict of interest.

## Footnotes

^1^http://www.mirbase.org
